# Covid-19 waste facemask conundrum: A facile way of utilization through fabricating composite material with unsaturated polyester resin and evaluation of its mechanical properties

**DOI:** 10.1016/j.heliyon.2022.e12197

**Published:** 2022-12-10

**Authors:** Mashrafi Bin Mobarak, Md. Sahadat Hossain, Fariha Chowdhury, Samina Ahmed

**Affiliations:** a Institute of Glass & Ceramic Research and Testing (IGCRT), Bangladesh Council of Scientific and Industrial Research (BCSIR), Dhaka 1205, Bangladesh; b Biomedical and Toxicological Research Institute (BTRI), Bangladesh Council of Scientific and Industrial Research (BCSIR), Dhaka 1205, Bangladesh; c BCSIR Laboratories Dhaka, Bangladesh Council of Scientific and Industrial Research (BCSIR), Dhaka 1205, Bangladesh

**Keywords:** COVID-19 pandemic, Face mask waste, Composite material, Hand lay-up technique, Mechanical properties

## Abstract

Since the outbreak of novel coronavirus (COVID-19), the use of personal protective equipment (PPE) has increased profusely. Among all the PPEs, face masks are the most picked ones by the mass people for protective purpose. This spawned extensive daily use of face masks and production of masks had to augment to keep up this booming demand. Such extensive use of face masks has resulted in a huge waste generation. Lack of proper disposal, waste management and waste recycling have already led this waste to pervade in the environment. In quest of finding a solution, here in this research, a composite material was fabricated utilizing waste face mask (WFM) with unsaturated polyester resin (UPR) and the mechanical properties were evaluated. The composites were fabricated by incorporating 1%, 2%, 3%, 4% and 5% WFM (by weight) within the UPR matrix in the shredded form following hand lay-up technique. Tensile properties, i.e., tensile strength (TS), tensile modulus (TM) and percentage elongation at break (% EB) as well as flexural properties, i.e., bending strength (BS) and bending modulus (BM) were evaluated for the fabricated composites. According to the results obtained, the 2% WFM loaded composites showed highest values of TS, TM, BS and BM which are 31.61 N/mm^2^, 1551.41 N/mm^2^, 66.53 N/mm^2^ and 4632.71 N/mm^2^ respectively. These values of 2% WFM loaded composite are 69.58%, 107.78%, 129.49% and 152% higher than the values of the control sample (UPR). Such results depict the successfulness of WFM's incorporation as a reinforcing material in the composite materials. Attenuated Total Reflectance-Fourier transform infrared spectroscopy (ATR-FTIR), scanning electron microscopy (SEM), water uptake and thickness swelling tests were also carried out for the fabricated composites. FTIR of the collected WFM revealed the fiber to be of polypropylene and the existing functional groups were also identified. The SEM images confirmed the proper adhesion of WFM and UPR in terms of mechanical bonding rather than chemical bonding. Water absorption and dimension change was investigated by water uptake and thickness swelling test. To sum up, the way we have utilized WFM as a reinforcing agent in a composite material, this could be a possible solution for the face mask's waste conundrum.

## Graphical abstract

**Figure:**
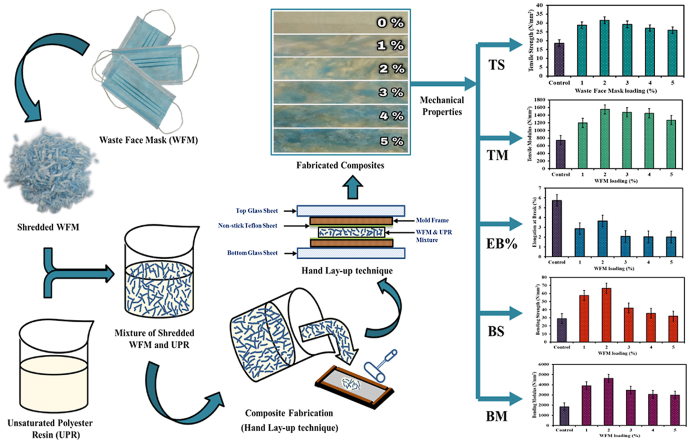

## 1 Introduction

The world has trembled with the outbreak of the severe acute respiratory syndrome or novel coronavirus (COVID-19) emerged from Wuhan, China back in December 2019 which was followed by SARS-CoV in 2003 and MERS-CoV in 2012 [1]. Till writing this manuscript, COVID-19 pandemic has caused demise to nearly 6.14 millions of people with 486 million confirmed cases and excruciating sufferings to countless people [2]. Right after the declaration as a pandemic form World Health Organization (WHO) [3, 4] many countries across the world have undertaken various measures to suppress the spread of this deadly virus including strict lockdown (staying at home) [5]. Strict lockdowns were withdrawn with the lessening of spread of this virus and other preventive measures like quarantine, isolation, social distancing, frequent washing of hands, avoiding mass gathering, use of personal protective equipment (PPE) (facial masks, gloves, face shields, wet wipes, gowns, shoes) etc. have been in effect according to the guidelines of WHO [6, 7, 8, 9, 10]. Among all the PPE's, face masks are the most prevalent since it is favored by both the health-care workers and mass people following the guidelines of WHO [11, 12]. The sudden use of face mask by the mass people with the hope of slowing down the transmission of coronavirus has not only led to global shortage [13, 14] but also raising concern for the colossal waste that is being produced [15, 16]. Many countries have boosted the production of disposable face mask and eased its way of distribution throughout the world [17]. This only adds more concern for the environmental pollution since improper disposal of facemask has been reported worldwide [18, 19, 20, 21, 22].

According to an international online survey, 19% of individuals throw away their disposable facemasks recklessly and even only 1% of disposable face masks by the world population would mean ∼10 million face masks (30,000–40,000 kg) discarded in the environment [23]. Since proper disposal of WFM aren't being ensured, scientists across the world have been trying to come up with alternative ideas for the utilization of such wastes. Asim et al. [24] reviewed the potential valorization techniques for discarded WFMs while Aragaw and Mekonnen et al. [25] have demonstrated a possible mitigation technique for waste-to-energy conversion using pyrolysis technique. In addition to that, Rehman et al. [26] utilized WFM with fat clay, making it an amalgamated binary admixture for better mechanical characteristics and WFM served as an additive in this regard. This whole idea of utilizing WFM into anything with potentiality might serve to mitigate the disposal problem.

Keeping this in mind, we have come up with a way to utilize WFM by making a composite material that will serve many purposes. According to Karbhari et al. [27], a composite material is a macroscopic combination of two or more distinct materials having a finite interface between them. Polymeric composite materials are usually fabricated by a combination of two phases; one is matrix which is normally organic polymer-based compound and the other one is the reinforcing material. The polymeric matrix can be either thermoplastic or thermosetting in nature. Most widely used thermoplastic matrixes are polyethylene, polypropylene (PP), poly vinyl chloride (PVC) while the most widely used thermosetting matrixes are polyester, epoxy and phenolic resins [28, 29]. The reinforcing materials that have been widely used are glass, Kevlar, aramid, carbon, natural fiber etc. [30, 31]. Such fabrication of composite using reinforcing material results in better mechanical properties than the matrix alone [32, 33]. The penchant of researchers has diverged from monolithic materials to fiber reinforced composite (FRCM) materials which is due to the nonpareil advantages of FRCM such as high strength to weight ratio, non-corrosive property and high fracture toughness [34, 35]. These advantages could serve the automotive and construction industries since they are always in quest of lighter and stronger materials of lower cost [36].

Here in this work, we have fabricated a composite material utilizing WFM and UPR. UPR is an extensively used polymeric matrix, mainly used in composite making [37, 38, 39, 40, 41], in flame retardation [42, 43, 44], in road construction [45], as corrosion resistant coating of steel [46], in furniture making [47, 48, 49] etc. owing to its noteworthy mechanical properties, dielectric properties, heat and chemical resistance, low cost, high gloss, ease of processing and good performance [50, 51]. The present study strives at preparation, characterization and evaluation of mechanical properties of the fabricated composite and utilization of WFM into a potential material was a perquisite.

## 2 Materials and methods

### 2.1 Materials

The polymeric matrix which is general purpose unsaturated polyester resin (UPR) and curing agent methyl ethyl ketone peroxide (MEKP) (Table 1) were purchased from Lucky Acrylic & Fiber, Dhaka, Bangladesh. MEKP functions as a hardener by cross linking the UPR matrix. These chemicals were used as received. The WFMs were collected from waste bins of different laboratories and households of close acquaintances. The chemical structure of UPR and MEKP is shown in Table 1 according to references.

**Table 1:** Structure of unsaturated polyester resin and methyl ethyl ketone peroxide.

Chemical Name	Unsaturated Polyester Resin	Methyl Ethyl Ketone Peroxide
Acronym	UPR	MEKP
Structure		
Reference	Recreated following: [52]	Recreated following: [53]

### 2.2 WFM processing

Among all the collected WFM, only the three layer surgical masks were sort out and kept secluded under sunlight for about 7 days [54] to eliminate any chances of initial contamination. After 7 days at seclusion, the masks were then subjected to washing using detergent-water followed by sun drying for 2 days. The WFMs were then cut in 10 cm by 10 cm dimension to eliminate elastic ear bands, nose clip and stitches and weighed. Again, the masks were sorted out based on the grams per square meter (gsm) and only 80 gsm three layered WFMs were selected for composite fabrication. The next step involves the manual shredding of WFM into fragments of roughly 1 mm by 10 mm and sorting of oversized fragments were done with manual visualization.

### 2.3 Composite fabrication

The shredded WFM fragments were mixed with UPR in a beaker and vigorous stirring was continued for 10–15 min to make sure of proper immixture of the components. This was followed by the addition of 1% (w/w) of curing agent (MEKP) into the mixture and vigorous stirring was continued for 2–3 min. The shredded WFM loading was varied to 1%, 2%, 3%, 4% and 5% by weight and UPR was added accordingly.

**Figure 1:**
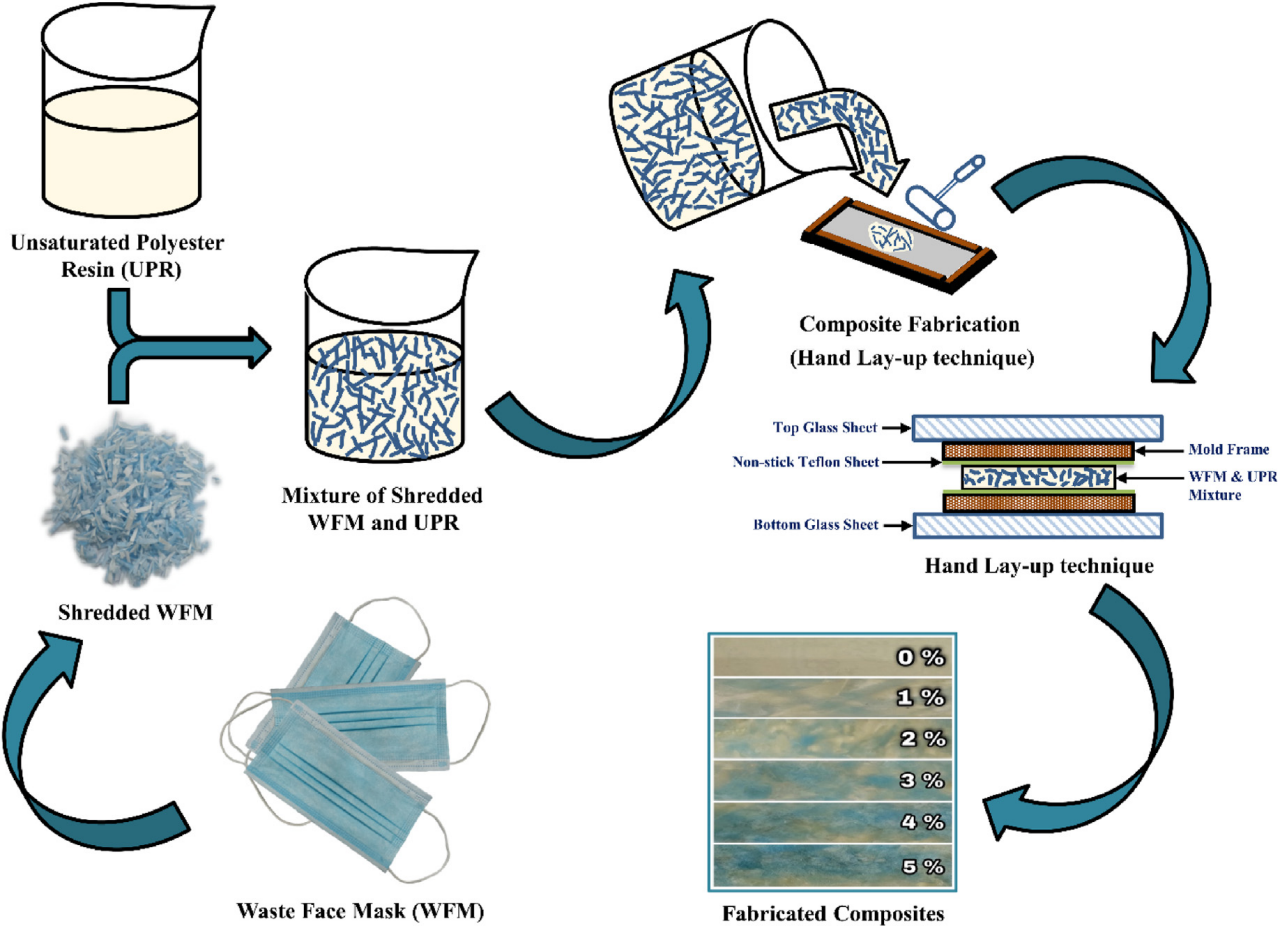
WFM and UPR based composite fabrication using hand lay-up technique.

The composite mixture was then poured into a pre-made mold frame of known thickness and the mold frame was placed over a glass sheet. Non-stick Teflon sheet was used beneath and above the mold frame to eliminate adhesion of the sample with the glass sheet. Another glass sheet was used on top of the mold frame making it a sandwich and composite was fabricated following the hand lay-up technique (Figure 1) [55]. The sample mixture was cured for 24 h inside a fume hood at temperature ∼25 °C. Finally, the composite was taken out of the mold frame, cut into appropriate dimension using a band saw and stored in air tight polyethylene bag.

### 2.4 Test methods

#### 2.4.1 Tensile and flexural test

The specimens for tensile and flexural tests were prepared in accordance to the ASTM D638-01 [56] and ASTM D790-10 [57] test methods. The tensile properties, i.e., tensile strength (TS), tensile modulus (TM) and percentage elongation at break (%EB) were evaluated by using a universal testing machine (Testometric M-500-30 KNCT, UK). The initial clamp separation of the machine was 20 mm and the cross-head speed was fixed at 20 mm/min. A 3000 kgf load cell was used in this regard and the tests were halted whenever failure occurred. The flexural tests, i.e. bending strength (BS) and bending modulus (BM) were also carried out with the aid of the same instrument. The cross-head speed was fixed at 10 mm/min and a 100 kgf load cell was used. The span distance of the three-point bending rig was 40 mm and the tests were halted whenever failure occurred. All the tests were carried out at the same conditions and the samples were kept in air tight polyethylene bag prior to any test. For better accuracy of the mechanical tests, five or more samples were evaluated for each test and the average value was considered. The resultant was a Time (sec) vs Force (N) curve showing values of Force @break (N) and Elongation @break (mm). The T.S, T.M and EB were calculated using the following Eqs. (1), (2), and (3).

(1) $$T.S=\frac{Force\text{@}break}{Area}$$

(2) $$\%\;E.B=\frac{Elongation\;\text{@}break\;}{Sample\;length}\times 100$$

(3) $$T.M=\frac{T.S}{E.B}$$

#### 2.4.2 Functional group analysis

The existing functional groups of the fabricated composites were detected by following fourier transform infrared spectroscopic (FTIR) analysis using IR Prestige-21 (Shimadzu Corporation, Japan) mounted with MIRacle-10 Single Reflection ATR (Attenuated Total Reflectance) accessory. Zinc selenide (ZnSe) prism plate was used for this single reflection. The analysis was done in the wavenumber range of 400–4000 cm^−1^ with a total number 30 scans per sample in the transmittance mode. Happ-Genzel apodization technique was implemented by the instrument where the resolution was 4 cm^−1^.

#### 2.4.3 Surface morphology analysis

The scanning electron microscopic (SEM) analysis was carried out to have a look into the surface morphology of the samples using Phenom Pro Desktop (Phenom 1481) at an accelerating voltage of 15 kv. Composite samples that were fractured during the tensile tests were selected in this regard and the images of the fractured surface were recorded.

#### 2.4.4 Water uptake test

The water uptake test was carried out following ASTM D570-98(2018) test method [58]. Initially, the composites were cut into small dimensions, cleaned to remove any dust or loose particle and weighed using an electrical analytical balance. Then they were submerged in 500 ml de-ionized (DI) water that was contained in a beaker and static motion and ambient temperature of fluid was ensured. The submergence was maintained for 30 days and the composite samples were withdrawn from the DI water periodically. After withdrawing for a brief time, the adherent water was removed using tissue paper, weighed and submerged again. This was continued up-to 30 days and the water uptake was calculated using the following Eq. (4) [59],

(4) $$Water\;Uptake\;\left(\%\right)=\frac{W_{wet}-W_{dry}}{W_{dry}}\times 100$$

Here, *W*_*wet*_ = weight of the wet sample withdrawn at a certain time and *W*_*dry*_ = weight of the initial dry sample.

#### 2.4.5 Thickness swelling test

The thickness swelling test was also carried out to investigate the dimensional changes that might occur when the composite samples are in contact with water. The procedure for this test was similar to that of the water uptake test but instead of weighing, the thicknesses of the samples were recorded. This was continued periodically for 30 days and the thickness was measured using a digital Vernier Caliper. The thickness swelling was calculated using the following Eq. (5) [60],

(5) $$Thickness\;swelling\;\left(\%\right)=\frac{T_{wet}-T_{dry}}{T_{dry}}\times 100$$

Here, *T*_*wet*_ = thickness of the wet sample withdrawn at a certain time and *T*_*dry*_ = thickness of the initial dry sample.

## 3 Results and discussion

### 3.1 Tensile tests

**Figure 2:**
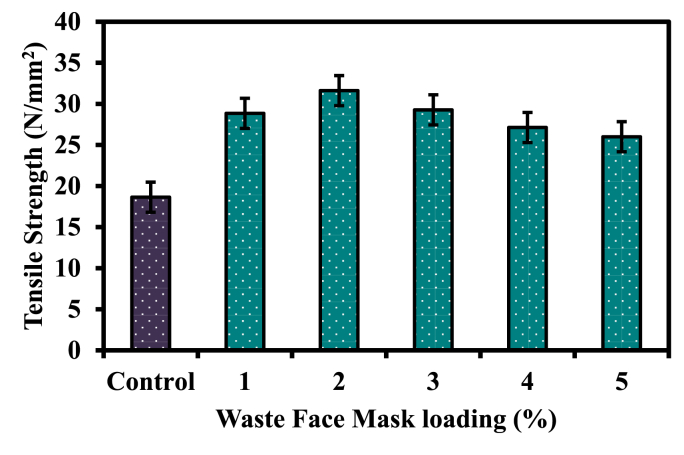
Tensile strength of UPR (control) and composites (UPR + WFM) with respect to WFM loading percentage.

The mechanical property is one of the decisive factors for the applicability of a composite in the desired field. Hence, the tensile and flexural tests were carried out in order to evaluate the mechanical properties of the composite. Figure 2 shows the TS of the UPR (control) and composites fabricated with UPR and WFM with respect to WFM loading percentage. The percentage of WFM loading was 1%, 2%, 3%, 4% and 5% by weight. The tensile tests were carried out at the optimal direction and only the samples that broke at the specific area were considered for further analysis. According to the data obtained, the control sample that is only made of UPR with the aid of curing agent, showed TS of 18.64 N/mm^2^. The addition of shredded WFM into the UPR evidently increased the TS but in different magnitude. At 2% WFM loading, TS was 31.61 N/mm^2^ which was highest among all the WFM loading composites and 69.58% higher than the control sample. Further addition of WFM into the composite didn't surpass the TS of 2% WFM reinforced composite but was definitely higher than the control sample. Such decrease in the TS might be due to the fact that upon the addition of WFM, the stress transfer between the matrix (UPR) and the reinforced fiber (WFM) got lessened which ultimately affected the TS of the composite. At 5% WFM loading, the TS was 25.99 N/mm^2^ which was 39.43% higher than the control sample but 21.62% less than the 2% WFM reinforced composite.

Since the TS of composite materials are dependent on the reinforcing material and amalgamation of WFM onto UPR resin has increased such, hence WFM evidently improved the mechanical property of the composite by acting as a reinforcing material.

**Figure 3:**
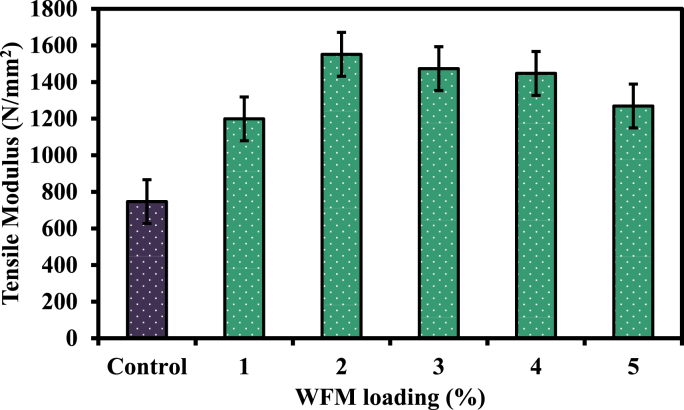
Tensile modulus of UPR (control) and composites (UPR + WFM) with respect to WFM loading percentage.

The tensile modulus (TM) is one of the features that describes the stiffness of the composite material. Likewise to that of the TS tests, the increase of WFM loading also increased the TM of the composite. Figure 3 shows the TM values of UPR (control) and the WFM reinforced composites at different WFM loading percentages. The TM values of the reinforced composites were higher than that of the control sample. TM achieved apex at 2% WFM loading and further addition decreased the TM up to 5% WFM loading. This was anticipated since the TS values also exerted the similar phenomena. The 2% WFM reinforced composite showed highest value (1551.41 N/mm^2^) which was 107.78% higher than the control sample (746.64 N/mm^2^).

**Figure 4:**
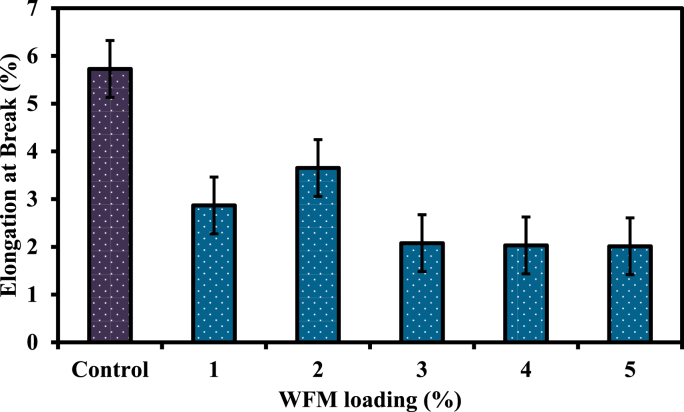
Percentage elongation at break of UPR (control) and composites (UPR + WFM) with respect to WFM loading percentage.

The control samples with the lowest values of TS and TM have shown the highest value of percentage elongation @break (5.73%); meaning that, it stretches to the highest compared to the reinforced samples as a percentage of its initial dimension until it breaks. The percentage elongation @break (%EB) was calculated using Eq. (3) and shown in Figure 4.

According to the results obtained, 2% WFM loaded composite showed the highest %EB (3.65%) among the reinforced composites which was 36.3% less than that of the control sample. Further increase in the WFM loading lessened the %EB and very close values were found for 3%, 4% and 5% loading (2.07, 2.03 and 2.01% respectively). When compared with the % EB values of control and WFM loaded composites, the addition of WFM into the UPR matrix decreased the %EB. This might be due to the fact that, upon the addition of WFM, the interaction between WFM-WFM increases while UPR-WFM interaction decreases. Higher the WFM-WFM interaction, lower the %EB.

### 3.2 Flexural tests

**Figure 5:**
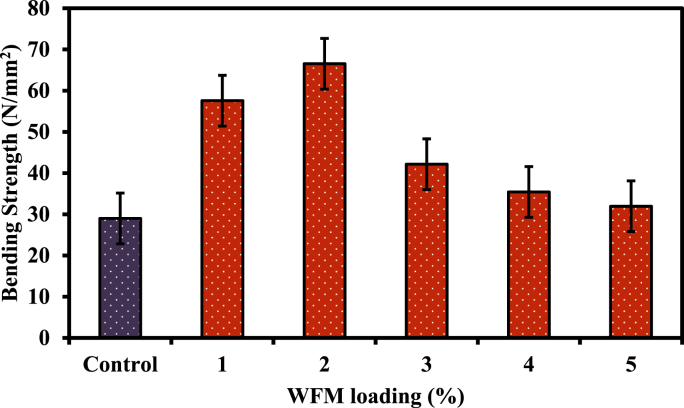
Bending strength of UPR (control) and composites (UPR + WFM) with respect to WFM loading percentage.

The flexural properties in terms of bending strength and bending modulus were also evaluated. The bending strength is an important factor for composite materials for demanding applications. It is defined as the ability of a composite material to resist bending deflection when energy is applied to the structure [61]. Figure 5 represents the bending strength of UPR (control) and the WFM reinforced composites at different WFM loading percentages.

**Figure 6:**
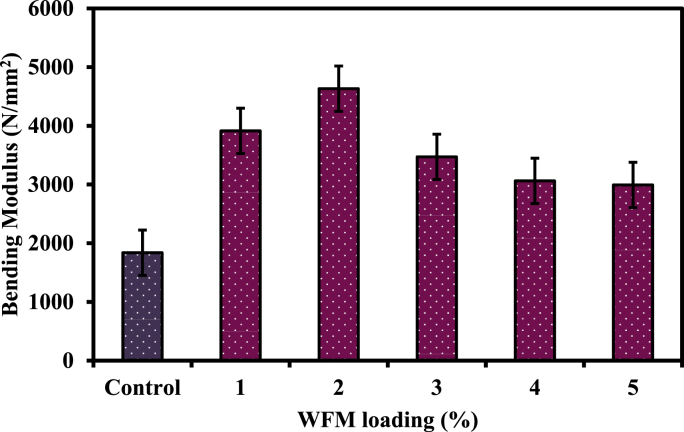
Bending modulus of UPR (control) and composites (UPR + WFM) with respect to WFM loading percentage.

Likewise to that of the tensile tests, flexural tests also transpired similar phenomena where the addition of WFM into the UPR matrix increased the BS. The control sample showed BS of 28.99 N/mm^2^ whereas 1% & 2% WFM reinforced composites showed 57.57 N/mm^2^ and 66.53 N/mm^2^ which were 98.58% and 129.49% greater than the control samples respectively. The BS of 2% WFM reinforced composite was the highest and further reinforcement lessened the BS. At higher loading percentages, the amount of matrix (UPR) is less and WFM is higher which invigorate the WFM-WFM interaction. On the contrary, the UPR-WFM interaction decreases which influences the flexural properties of the composite and begets lower values of BS. The phenomenon is similar for the bending modulus of the control and the WFM reinforced composites which has been shown in Figure 6.

The BM illustrates the stress-strain ratio of the control and composite materials within their elastic region [62]. The 2% WFM reinforced composite with highest BS also had the highest BM (4632.71 N/mm^2^), 152% higher than that of the control sample (1838.36 N/mm^2^). Such significant modification of the flexural properties does indicate the succession of WFM reinforcement within the UPR matrix.

### 3.3 Functional group analysis

**Figure 7:**
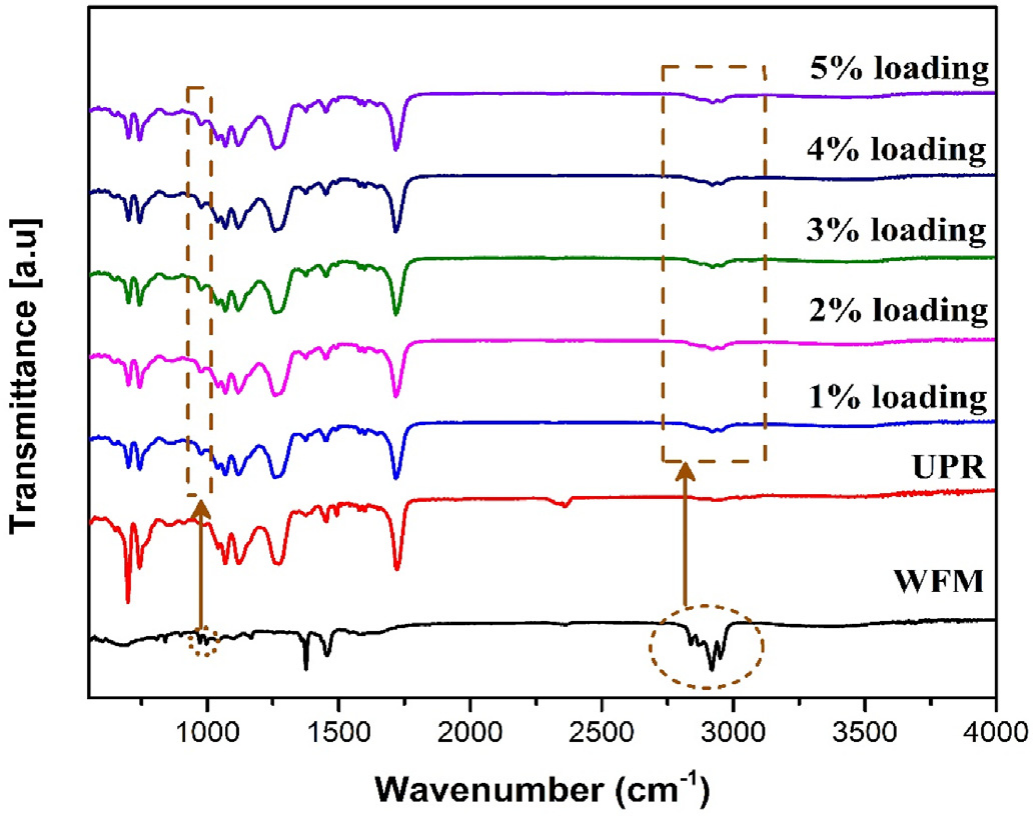
ATR-FTIR spectra of WFM, UPR, 1%, 2%, 3%, 4% and 5% WFM loaded composites.

To get hold of the existing functional groups of the WFM, UPR and the WFM reinforced composites, ATR-FTIR spectrum was recorded which has been shown in Figure 7. Generally, face masks are made of polypropylene, polyethylene, polystyrene, polyester, polycarbonate, polyurethane, polyacrylonitrile [63]. The classical 3-ply face masks are comprised of polypropylene or polyester that goes through melt blowing in order to make the non-woven fabrics of the face mask [64]. The ATR-FTIR spectrum that has been recorded for the outer layer of the WFM indicates its formation with polypropylene fibers [65, 66]. The symmetry deformation vibration peak of the methylene group on the aliphatic hydrocarbons appears at 1456 cm^−1^ (moderate intensity) and the methyl group vibration peak appears at 1375 cm^−1^ which are in compliance with the data reported in previous literature [25, 67].

For the spectrum of WFM, between the wavenumber 2800 cm^−1^ and 3000 cm^−1^, four adjacent peaks were observed. For the symmetric stretching vibrations of CH_2_ and CH_3_, two peaks were observed at 2918 cm^−1^ and 2949 cm^−1^. On the other hand, peaks at 2866 cm^−1^ and 2837 cm^−1^ corresponds to the asymmetric stretching vibrations of CH_2_ and CH_3_ [68,69]. According to the findings of Morent et al., the low intense peak at 1166 cm^−1^ can be assigned to CH_3_ asymmetric rocking, C–C asymmetric stretching and C–H wagging vibrations. The asymmetric rocking vibration exerted peak at the wavenumber of 997 cm^−1^. At the wavenumbers of 972 cm^−1^ and 898 cm^−1^ two peaks appeared for the vibration of C–C asymmetric stretching and CH_3_ asymmetric rocking and symmetric stretching. The rocking vibration of CH_2_ was confirmed from the peaks of 840 and 808 cm^−1^ and similar peaks were also reported [70].

The peak from 3200 cm^−1^ to 3600 cm^−1^ was identified which carried good evidence of the stretching vibration of –OH groups in the spectrum of polymer matrix (UPR). Less intense peaks at 2856 cm^−1^ to 2929 cm^−1^ were originated form the symmetric stretching vibration of C–H. One of the most intense peaks was appeared for the presence of carbonyl group (C=O) at the wavenumber of 1720 cm^−1^ and similar peak was reported elsewhere [37, 71, 72]. The stretching vibrations of aromatic C=C exerted peaks at 1598, 1577 and 1492 cm^−1^. Symmetric and asymmetric bending vibrations of the methyl (CH_3_) group were noticed at 1452 cm^−1^ and 1375 cm^−1.^ The peaks around the fingerprint region (1273 cm^−1^, 1120 cm^−1^, 1068, cm^−1^ and 1041 cm^−1^) were identified for the existence of stretching vibrations of C–O group for ester molecules. Strong peaks at 698 cm^−1^, 742 cm^−1^ and 989 cm^−1^ were referred to the out-of-plane bending vibration of =C–H group in aromatic ring, vibrations of substituted aromatic ring and C–H out-of-plane bending vibration of trans –CH=CH– and similar peaks were documented elsewhere [73]. To summarize, all the vibrational assignments of the WFM and UPR has been shown in Table 2.

**Table 2:** Peak position and assignments of ATR-FTIR absorption bands of WFM and UPR.

Sample Name	Peak Position (Wavenumber, cm^−1^)	Assignments
WFM	2949	CH_3_ symmetric stretching
	2918	CH_2_ symmetric stretching
	2866	CH_3_ asymmetric stretching
	2837	CH_2_ asymmetric stretching
	1456	CH_2_ symmetry deformation
	1375	CH_3_ bending
	1166	C–C asymmetric stretching CH_3_ asymmetric rocking C–H wagging
	997	CH_3_ asymmetric rocking
	972	CH_3_ asymmetric rocking C–C asymmetric stretching
	898	CH_3_ asymmetric rocking C–C asymmetric and symmetric stretching
	840	CH_2_ rocking
	808	CH_2_ rocking
UPR	3200–3600	–OH stretching
	2856–2929	C–H symmetric stretching
	1720	carbonyl group (C=O) (ester linkage)
	1598, 1577 and 1492	C=C stretching within the aromatic ring
	1452	CH_3_ asymmetric bending
	1375	CH_3_ symmetric bending
	1273, 1120, 1068 and 1041	C–O stretching (ester)
	989	C–H out-of-plane bending of trans –CH=CH–
	742	=C–H out-of-plane bending of aromatic ring
	698	=C–H out-of-plane bending in singly substituted aromatic ring

Abbreviations: WFM = Waste Face Mask; UPR = Unsaturated Polyester Resin.

The four significant peaks of WFM 2949 cm^−1^, 2918 cm^−1^, 2866 cm^−1^ and 2837 cm^−1^ can also be observed in the FTIR spectra of 1%–5% WFM loaded composites which indicate the incorporation of WFM within the UPR matrix. Also, peaks at 997 cm^−1^ and 972 cm^−1^ can be observed within the WFM reinforced composites but at different intensities depending on the WFM loading.

### 3.4 Surface morphology analysis

**Figure 8:**
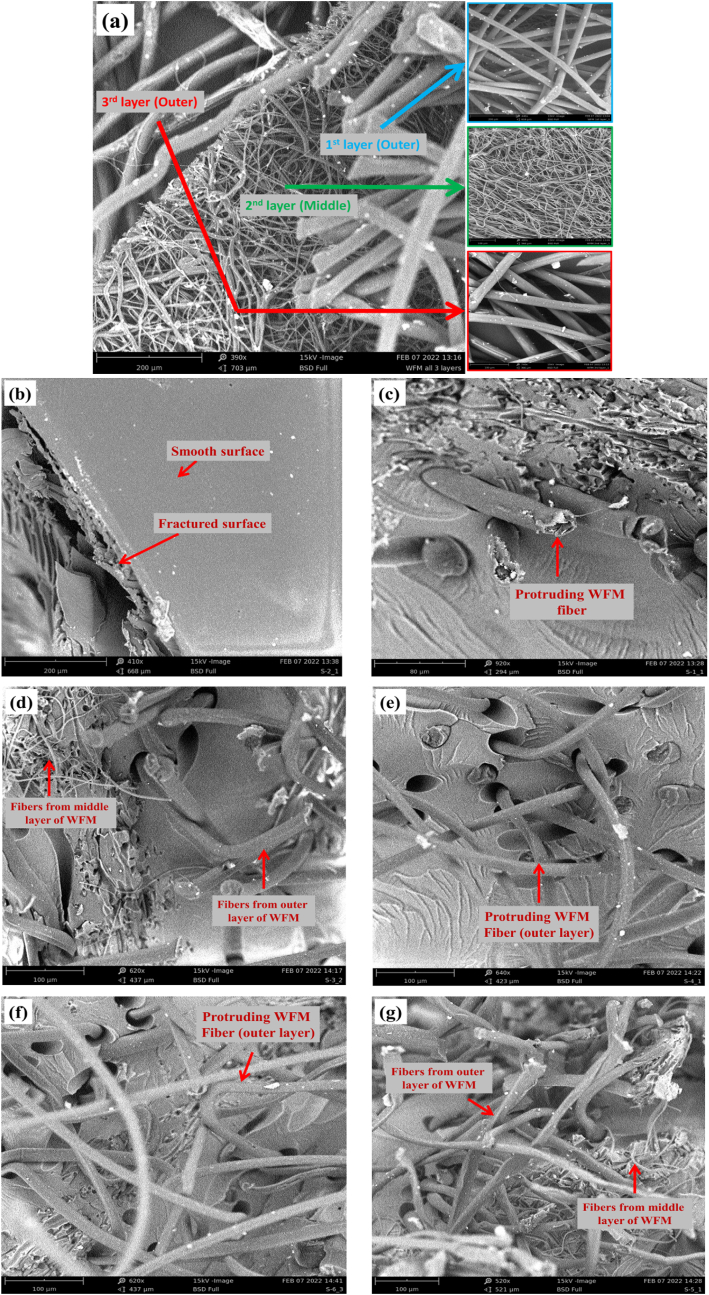
SEM images of (a) typical WFM; fractured surfaces of (b) control (UPR), (c) 1%, (d) 2%, (e) 3%, (f) 4% and (g) 5% WFM loaded composites.

The scanning electron micrographs of the WFM as well as the fractured surfaces of WFM loaded composites have been captured in order to investigate the interaction of the fibers with the polymer matrix. Figure 8(a) shows the SEM image of a typical WFM which is comprised of three layers. The outer layer (1st layer) fibers are much dense than the fibers of outer layer (3rd layer). Fibers of the middle layer (2nd layer) are smaller in diameter and much dense than both the outer layers (1st and 3rd layer). Such fibril arrangement serves as a filtration system against dust or contaminant particles. The diameter of the fibers has been measured using imageJ software based on the SEM image. The average fiber diameter of the outer layer (1st layer), middle layer (2nd layer) and the outer layer (3rd layer) has been found to be 29.60 μm, 3.20 μm and 28.54 μm respectively. Figure 8(b) represents the SEM image of control which is the UPR matrix. The undisrupted surface of the control sample is smooth with no perforations or voids which is also confirmed by looking into the fractured surface. Figure 8(c)–(g) represents the fractured surfaces of the 1%–5% WFM loaded composites. The presence of fibers at the fractured surface is more apparent with increasing fiber loading. Very few holes can be seen at the fractured surfaces of the composites at lower WFM loading, indicating that the WFM fibers were strongly agglutinated with the UPR matrix which resulted in low pull out of the fibers. But slightly different scenario was observed for the composites with higher WFM loading.

This is due to the fact that, at higher WFM loading, the amount of UPR is decreased and this results in less agglutination between the fiber and UPR. It seems like an over-crowdedness or over-saturation of WFM fibers in the matrix and this consequently affects the mechanical property of the composites. Both the outer layer and middle layer WFM fibers are present at the fractured surfaces of the fabricated composites.

### 3.5 Water uptake test

**Figure 9:**
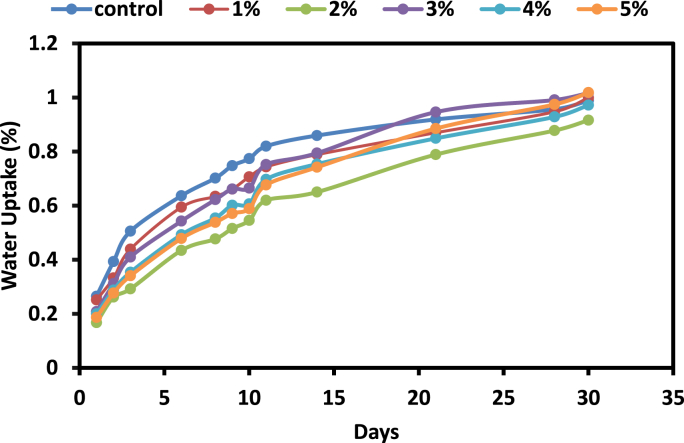
Water uptake of control (UPR) and WFM loaded composites.

The percentage of water uptake of the composite samples for a period of 30 days has been shown in Figure 9. The test was carried out with four samples for each WFM loaded composites and the control sample from which an average value is obtained. According to the data obtained, the percentage of water uptake was found to be increased with increasing immersion time. Highest percentage of water uptake (1.0182 %) was found for 5% WFM loaded composites and the lowest percentage (0.9161 %) was found for 2% WFM loaded composites after 30 days of immersion.

It has been noticed that within the 30 days of test period, the percentage of water uptake didn't reach to any constant value and hence depicts the slowness of water uptake by the WFM loaded composites. Within the 15 days period, the rate increased swiftly which slowed down afterwards. An increase in WFM loading didn't necessarily increased the rate of water uptake. This may be due to a lot of reasons since the rate of water uptake greatly depends on temperature, time of immersion, loading of the WFM, orientation of WFM fiber, area of exposed surface, reaction between water and matrix, interfacial bonding, surface protection and voids, diffusivity etc. [74].

### 3.6 Thickness swelling test

**Figure 10:**
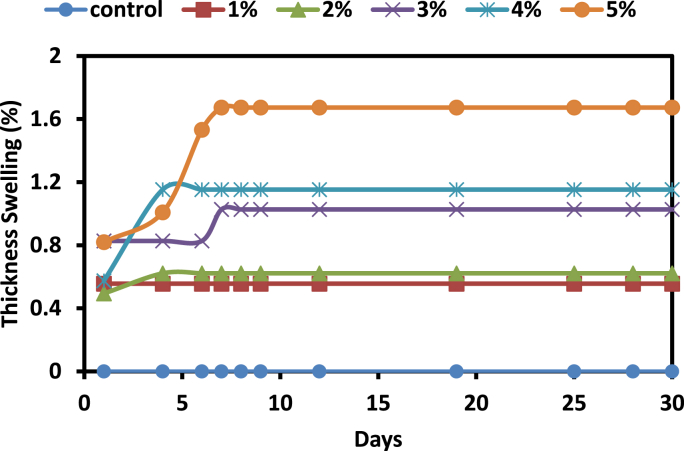
Thickness swelling of control (UPR) and WFM loaded composites.

The results of thickness swelling test have been shown in Figure 10. This test was also carried out for a 30 days’ time period and thickness was measured periodically. The significance of this test is related to the change of dimension of the composite samples upon water uptake at certain period of time. According to the data obtained, the effect of thickness swelling is less compared to the water uptake percentage. The control sample (UPR) showed no sign of thickness swelling although it uptakes 0.988% water after 30 days. Highest percentage of swelling (1.673%) was observed for 5% WFM loaded composites where the thickness increased until the 7th day and remained constant up to 30th day. The 1% WFM loaded composite showed thickness swelling only for the first day and the rest was constant. The 2%, 3% and 4% WFM loaded composites showed initial thickness swelling up to 4th, 7th and 4th day respectively and rest days were at constant values.

The swelling effect was not that much significant for the WFM loaded composites. One possible reason might be due to the softening of composite under the influence of water that reduces the rigidity. In addition to that, the WFM fibers were shredded to a certain size rather than a continuous thread. With such case, the absorbed water by protruding WFM fibers at the boundary wall were unable to move inside the material significantly and thus low thickness swelling resulted. Since no WFM fibers are present in the control sample, thickness swelling wasn't observed as a repercussion. The 5% WFM loaded composite has the highest amount of WFM fiber and these fibers are close enough to make an inter-connection; this aided in greater water uptake as well as swelling of the composite material.

## 4 Conclusion

Since its emergence, the cataclysmic corona virus (covid-19) has affected our daily life as well as the environment with wastes that are most likely to persist for at least couple hundred years. The waste generation associated with PPE, especially the waste face mask is already a perturbation for the concerned society as well as for the researchers trying to find an immediate solution. Here in this research, waste face masks have been utilized by making composite material using unsaturated polyester resin. The key findings of this research include.

i. Inclusion of WFM into UPR matrix has evidently increased the mechanical properties of the composites which indicate its potential applicability as a reinforcing material.
ii. The 2% WFM loaded composite exhibits highest tensile and flexural properties compared to control, 1%, 3%, 4% and 5% WFM loaded composites. This depicts the amount of WFM that is in better interaction with the UPR matrix which ultimately shows better physical properties.
iii. The functional group analysis confirmed the fibers of the collected WFM to be of polypropylene. The existing functional groups of the UPR matrix have also been confirmed. The inclusion of WFM within the UPR matrix was also confirmed by the FTIR analysis.
iv. SEM analysis confirmed strong adherence of the WFM fibers with the UPR matrix as well as very few fiber pull outs. Three different sizes of WFM fibers were confirmed within the fractured surface of the composites.
v. Water uptake and thickness swelling tests revealed the water absorption capacity and consequential dimensional change within the composite materials. The 5% WFM loaded composite showed highest thickness swelling and water uptake due to highest amount of shredded WFM in close proximity and making an inter-connection for water to enter.

According to the findings of this research, it can easily be concluded that, waste face mask can be utilized in making composite material with better mechanical properties and hence, have potential applicability in making household articles, containers, roofing, door panels, automotive structure, insulation boards, door/window frames etc. [30, 75]. The idea of utilization of a waste material that is associated with the covid-19 outbreak will allow researchers to find solutions of this waste conundrum. There is scope for further investigation with different types of face masks as well as other PPEs which will also be crucial in covid-19 breed waste utilization.

## Declarations

### Author contribution statement

Mashrafi Bin Mobarak: Performed the experiments; Analyzed and interpreted the data; Wrote the paper.

Md. Sahadat Hossain: Conceived and designed the experiments; Analyzed and interpreted the data.

Fariha Chowdhury: Performed the experiments; Analyzed and interpreted the data.

Samina Ahmed: Conceived and designed the experiments; Contributed reagents, materials, analysis tools or data.

### Funding statement

This work was supported by IGCRT, BCSIR (R&D approval ref. 39.02.0000.011.14.134.2021/900; Date: 30/12/2021).

### Data availability statement

Data included in article/supplementary material/referenced in article.

### Declaration of interests statement

The authors declare no competing interests.

### Additional information

No additional information is available for this paper.
